# Effect of Dielectric Barrier Discharge (DBD) Treatment on the Dielectric Properties of Poly(vinylidene fluoride)(PVDF)-Based Copolymer

**DOI:** 10.3390/polym12061370

**Published:** 2020-06-18

**Authors:** Jie Liu, Yang Zhou, Kewang Yi, Shihai Zhang, Tao Shao, Cheng Zhang, Baojin Chu

**Affiliations:** 1 CAS Key Laboratory of Materials for Energy Conversion and Department of Materials Science and Engineering, University of Science and Technology of China, Hefei 230026, China; lj626@mail.ustc.edu.cn (J.L.); zhengzezhiqiang@126.com (Y.Z.); kwyi@mail.ustc.edu.cn (K.Y.); 2 Strategic Polymer Sciences, Inc., 200 Innovation Boulevard, State College, PA 16803, USA; energy@polyktech.com; 3 Institute of Electrical Engineering, Chinese Academy of Sciences, Beijing 100190, China; st@mail.iee.ac.cn (T.S.); zhangcheng@mail.iee.ac.cn (C.Z.)

**Keywords:** dielectric barrier discharge, polymers, dielectric breakdown strength, dielectric properties

## Abstract

Understanding the mechanism of dielectric breakdown is important for improving the breakdown field of a polymer. In this work, dielectric barrier discharge (DBD) treatment was applied to one surface of P(VDF-CTFE) (vinylidene fluoride-chlorotrifluoroethylene) film, and the dielectric properties of the film were studied. When the treated surface was connected to the high potential side of the power source for the breakdown test, the breakdown field of the treated film was significantly reduced compared to that of the pristine film. Based on the characterization results for the surface chemistry and morphology, it was proposed that the phenomenon was caused by the combined effects of hole injection from the metal electrode and the damage of polymer chains near the surface of the polymer film after the DBD treatment process.

## 1. Introduction

There is growing demand for dielectric materials with high energy density for capacitor applications. The energy density of a dielectric material is related to the dielectric constants and dielectric breakdown strength of the material [1,2]. Dielectric polymers have the advantages of being light-weight, easy to process and low cost and of having a high breakdown strength, and the materials are an important class of materials for energy storage applications [3,4,5,6]. Poly(vinylidene fluoride) (PVDF)-based ferroelectric polymers have higher dielectric constants than many dielectric polymers and a high energy density has been achieved in this type of material [7,8,9]. For example, an energy density higher than 25 J/cm^3^ was obtained in P(VDF-CTFE) copolymers, which is promising for capacitor applications [9,10,11]. Because breakdown strength is a parameter that determines the energy density of polymers, understanding the mechanism of the breakdown strength is important for improving the breakdown strength and energy density. For solid dielectrics, different dielectric breakdown mechanisms have been proposed, including charge injection, avalanche mechanism, electromechanical breakdown and thermal mechanism [12,13]. The breakdown mechanisms of PVDF-based materials have also been studied. For example, Chen et al. found that hole injection is a major cause for the breakdown of PVDF-based terpolymers [14]. In another study, it was proposed that the electromechanical breakdown mechanism is responsible for the dielectric breakdown of P(VDF-CTFE) [15]. Therefore, the key mechanism that is responsible for the dielectric breakdown is not completely understood and further study is needed to understand the dielectric breakdown mechanism of PVDF-based polymers.

**Figure 1:**
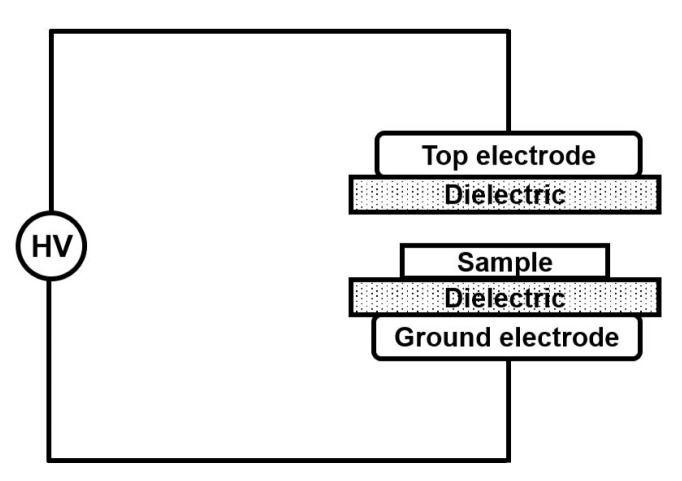
A schematic of dielectric barrier discharge (DBD). The left part of HV (High voltage source) represents a compact microsecond-pulse generator.

Dielectric barrier discharge (DBD) treatment is a high voltage gas discharge method with non-thermal plasma at atmospheric pressure [16,17]. In this method, a dielectric barrier is used in the discharge gap, by which the electric current and spark formation can be suppressed, as schematically shown in Figure 1. It was found that the DBD arises from a massive amount of individual tiny breakdown channels, which are considered as microdischarges [18]. Due to the lack of sparks, dielectric barrier discharge is also called silent discharge, and it is widely applied to modify polymer surfaces to improve physical and chemical properties of polymers, such as wettability, printability and adhesion [19,20,21]. For dielectric applications, in order to increase the surface insulation performance of polymers, DBD has been used to modify the surface properties of epoxy material, as reported in a recent work [22]. Liu et al. used DBD to modify the surface of PET (polyethylene terephthalate) polymer and it was found that the modification had little effect on the dielectric properties [23]. Nevertheless, there are few studies related to the effect of DBD on dielectric properties of polymers.

In this work, DBD was used to modify the surface of one side of P(VDF-CTFE) (15 wt % CTFE) film to study the effect of the treatment on the dielectric properties and breakdown strength of polymers. We found that the breakdown strength was reduced when the treated surface was connected to the high potential end of the power supply for the breakdown test. We propose that the breakdown of the film is initiated from the high potential side of the film. The damage of the surface by the DBD treatment and hole injection are two major factors causing the reduction in the breakdown strength. This work provides an in-depth understanding of the breakdown mechanism of the P(VDF-CTFE) polymer, which is important for the improvement of energy storage performance of the polymer.

## 2. Materials and Methods 

P(VDF-CTFE) (15 wt % CTFE) blown films (around 10 µm thick) were provided by PolyK Technologies (State College, PA, USA). X-ray diffraction (XRD) patterns of the polymers were obtained using a SmartLab TM9kw diffractometer (Rigaku, Tokyo, Japan). The X-ray diffraction used Cu-Kα radiation and the wavelength was 0.15406 nm. The Fourier-transform infrared spectra (with attenuated total reflectance, ATR-FTIR) of the samples were obtained using a Thermo Fisher-Nicolet 6700 FTIR Spectrometer (Nicolet, Madison, WI, USA) with a diamond probe. The surface morphology of all samples was characterized by a SU8220 field emission scanning electron microscope (FE-SEM) (Hitachi Ltd, Hitachi, Japan). X-ray Photoelectron Spectroscopy (XPS) was performed by ESCALAB 250 (Thermo-VG Scientific, Waltham, MA, USA), which was calibrated with reference to C1s at 284.8 eV to eliminate interference from the charging. Atomic Force Microscope (AFM) measurement was taken with a DI Innova scanning probe microscope (Veeco Inc, Plainview, NY, USA). The AFM measurement used tapping mode with a silicon probe. To test the dielectric constant, polarization-electric field (P-E) hysteresis loops and breakdown strength, gold electrodes were coated on both sides of the film via a DC sputtering method using a sputter coater (EMS150T, Electron Microscopy Sciences, Hatfield, PA, USA). The dielectric properties of the polymer samples as a function of temperature were measured using an LCR meter (Agilent E4980A, Agilent Technologies, Santa Clara, CA, USA) equipped with a temperature chamber. The P-E loops were measured using a modified Sawyer-Tower circuit (PolyK Technologies, State College, PA, USA) and the test frequency was 10 Hz. The breakdown strength was measured by a system consisting of a controlling circuit and a high voltage amplifier (10/10B-HS, Trek, Lockport, PA, USA) with a 200 V/s ramping rate. For the dielectric barrier discharge (DBD) treatment, the films were placed on the ground electrode, as shown in Figure 1 and a high voltage was applied to the electrodes by a compact microsecond-pulse generator with a semiconductor opening switch (SOS) [24]. The polymer films were treated at 8 kV for 60 s, 10 kV for 15 s and 60 s with a 1 mm gap distance to generate a filamentary discharging mode. The treatment performed at 8 kV did not generate a stable dielectric barrier discharge. Only one side of each film was treated by the DBD.

## 3. Results and Discussion

**Figure 2:**
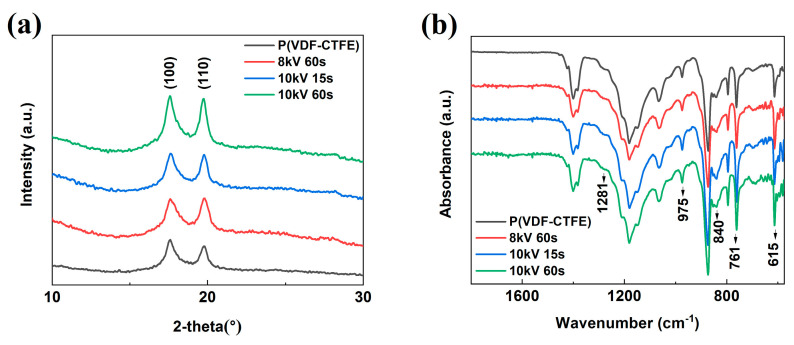
(**a**) XRD patterns of P(VDF-CTFE) and films modified by DBD under different conditions. (**b**) ATR-FTIR spectra of P(VDF-CTFE) and films modified under different conditions.

**Figure 3:**
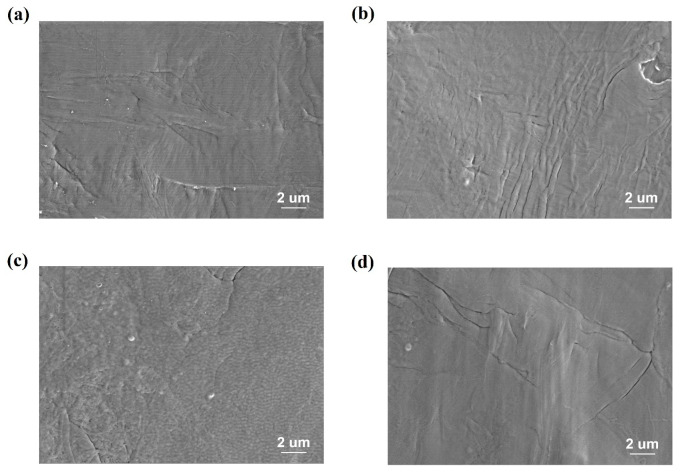
The surface SEM images of (**a**) P(VDF-CTFE). (**b**) The film treated at 8 kV for 60 s. (**c**) The film treated at 10 kV for 15 s. (**d**) The film treated at 10 kV for 60 s.

Figure 2a shows the XRD patterns of the treated and untreated films. The crystalline phase of the films is determined to be an α-like phase, as manifested by the (100) and (110) peaks near 20° [25,26]. All the treated films have these two peaks without any shift or obvious change of peak intensity ratio, indicating that the DBD treatment has no discernible effect on the crystal structure. The results of ATR-FTIR spectra shown in Figure 2b are consistent with the XRD study. The peaks around 615, 761 and 975 cm^−1^ are assigned to the TGTG’ conformation which represents the *α* phase. The weak peaks around 840 and 1281 cm^−1^ correspond to the T_m>4_ and T_3_G conformation. These results indicate that the crystalline phase of all films is mainly *α* phase. No obvious change in microstructure after DBD treatment can be observed from FTIR data [27,28]. The surface morphology of the films before and after DBD treatment was observed by SEM and the surface images are shown in Figure 3. From Figure 3, we can see that no obvious change of surface morphology can be observed in micrometer scale after the DBD treatment.

**Figure 4:**
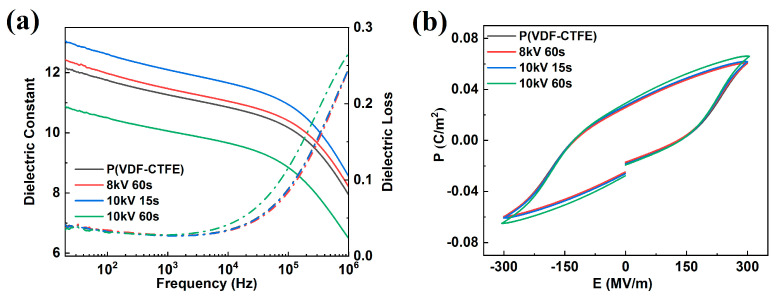
Characterization of the low-field and high-field dielectric properties of films. (**a**) The dielectric constant (solid lines) and loss (dotted lines) at different frequencies. (**b**) The polarization-electric field (P-E) loop at 300 MV/m.

Figure 4a shows the frequency dependences of the dielectric properties of the pristine film and the DBD-treated films at room temperature. As shown in Figure 4a, the dielectric constant of the untreated film is ~12. The treatment at 8 kV for 60 s and 10 kV for 15 s slightly increase the dielectric constant without changing the dielectric loss. The dielectric constant of the film treated at 10 kV 60 s was reduced compared with pristine film. Additionally, the dielectric loss is slightly increased, especially at a high temperature, which probably occurs because of over-exposure in the DBD treatments and damage to the film. The P-E loops of the treated and untreated films measured at 300 MV/m are presented in Figure 4b and show that the DBD treatment does not significantly modify the polarization response under a high field.

**Figure 5:**
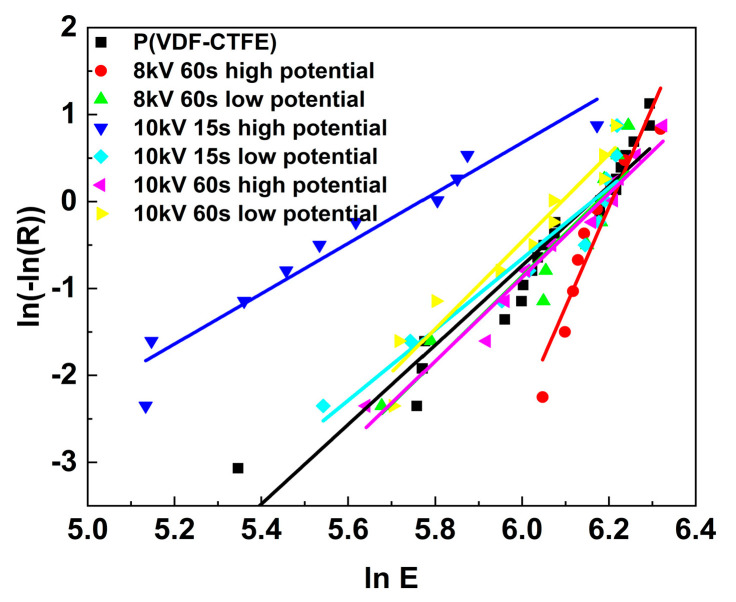
Weibull analysis of the dielectric breakdown strength data of the P(VDF-CTFE) film and the DBD-treated films. For the treated films, breakdown tests were performed with the treated surface connected to either the high potential or low potential side of the power supply.

The dielectric breakdown field of the untreated and treated P(VDF-CTFE) films was measured and the results were analyzed by Weibull distribution, as shown in Figure 5 [14]. The characteristic breakdown field of the samples is summarized in Table 1. For P(VDF-CTFE), the dielectric breakdown strength is about 473.97 MV/m, which is consistent with the results reported in the literature [10,29]. For the DBD-treated films, there is an untreated surface and a treated one. The results shown in Table 1 indicate that the breakdown field of the DBD treated film is dependent on the connection of the two surfaces with the power supply for the breakdown test. The film treated at 8 kV for 60 s has a dielectric breakdown strength similar to that of the untreated film regardless of which surface is connected to the high potential side of the power supply. For the films treated at 10 kV for 15 s and 60 s, if the treated surface is connected to the low potential side of the power supply, the dielectric breakdown strength is almost the same as that of the untreated film. However, if the treated surface is connected to the high potential side, the breakdown field is significantly reduced by at least 154 MV/m for the 10 kV 15 s samples and 32 MV/m for the 10 kV 60 s samples.

**Figure 6:**
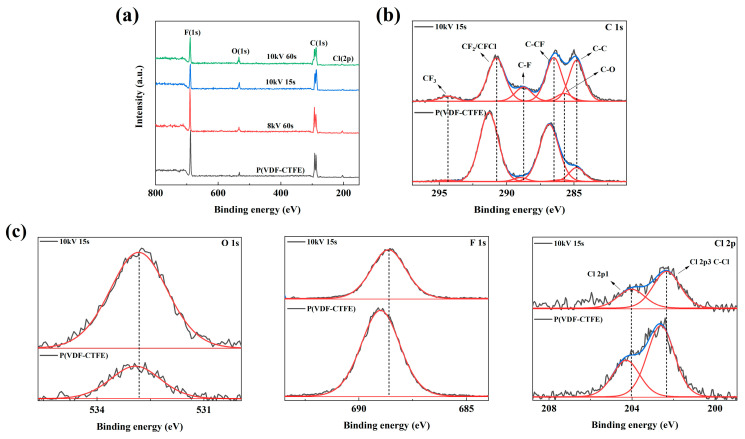
(**a**) The XPS survey scan of P(VDF-CTFE) and the films treated under different conditions. (**b**) Carbon (C 1s) spectra of P(VDF-CTFE) and the film treated at 10 kV for 15 s. (**c**) Oxygen (O 1s), fluorine (F 1s) and chlorine (Cl 2p) spectra of P(VDF-CTFE) and the film treated at 10 kV for 15 s.

According to the XRD, FTIR-ATR spectra and dielectric data, it is obvious that the lowering of the breakdown strength by connecting the DBD-treated surface of the P(VDF-CTFE) film to the high potential end of the power supply is not caused by the change in the microstructure and properties of bulk, but is related to the modification of the surface of the films by DBD treatments. Because the results of XRD, FTIR and dielectric properties primarily reflect the bulk properties of the polymer, to investigate the effect of DBD treatment on the surface properties, XPS and AFM tests were performed [30,31]. XPS measurement was conducted to characterize the change of surface chemistry of the films after the films were treated by DBD. The XPS spectra of the untreated film and the film treated under different conditions is shown in Figure 6a, and the content of different elements in the films is calculated and summarized in Table 2 [32,33]. From Figure 6a, the F peak and Cl peak of the 8 kV 60 s sample are similar to the P(VDF-CTFE), while for the samples treated at 10 kV, they are weakened. Meanwhile, the O peak increases for the samples treated at 10 kV. From Table 2, we can see that the surface composition of the film changes significantly after DBD treatment. The content of oxygen increases greatly at the expense of F and Cl elements. Figure 6b shows the C1s spectra of P(VDF-CTFE) and film treated at 10 kV for 15 s. The C1s band can be deconvoluated into six peaks, which can be assigned to C–C (284.8 eV), C–O (285.94 eV), C–CF (286.85 eV), C–F (288.99 eV), CF_2_/CFCl (291.31 eV) and CF_3_ (294.48 eV). The peaks of C–O, C–CF, C–F, CF_2_/CFCl and CF_3_ shift to lower binding energy, which indicates the reduction of strong electron-withdrawing groups. This may be caused by some positive defects due to the defluorination and dechlorination under high voltage [32,34]. From Figure 6c, the peaks for O 1s, F 1s and Cl 2p also shift to a lower binding energy. Therefore, the XPS results suggest that the DBD treatment causes defluorination and dechlorination of surface and induces the damage of polymer chains on the surface [35,36]. At the same time, the surface is oxidized, and the oxidation may chemically degrade the polymer chains [37,38].

**Figure 7:**
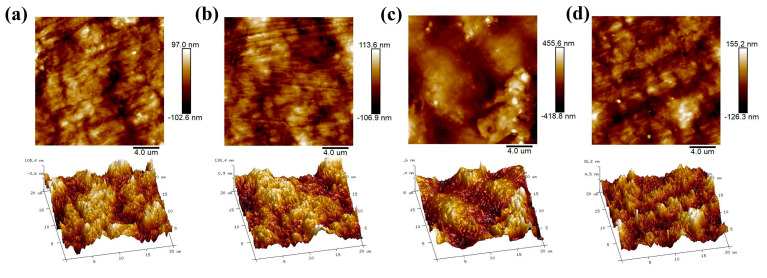
The surface morphology and roughness characterized by Atomic Force Microscope (AFM). (**a**) Untreated P(VDF-CTFE) film. (**b**) The film treated at 8 kV for 60 s. (**c**) The film treated at 10 kV for 15 s. (**d**) The film treated at 10 kV for 60 s.

In addition to the change in surface chemistry, DBD treatment has a significant effect on the morphology of the surface of the film at nanoscale. The surface morphology of the treated and untreated films was characterized by AFM and is shown in Figure 7. In Figure 7, for each sample, plane and three-dimensional views of the surface were presented. The surface roughness (Ra) of the films is summarized in Table 3 [38,39,40,41]. As shown in Figure 7, the DBD treatment increases the surface roughness of the polymer film, especially in the film treated at 10 kV for 15 s. The surface roughness of untreated film is approximately 22.4 nm, and increases to 87.7 nm after the film is treated at 10 kV for 15 s. Further treatment at 10 kV for 60 s seems to smooth over the surface and the roughness is reduced. Both the XPS and AFM results suggest that the DBD treatment can chemically produce appreciable damage on the film surface.

For dielectric breakdown, the thermal and electromechanical mechanisms are primarily dependent on the bulk properties of the polymer. However, the XRD, FTIR and dielectric results indicate that the bulk properties were not changed by the DBD treatment. The treatment only changed the surface of the polymer film. Therefore, it can be concluded that the observed change of breakdown behavior in the treated film is caused by the change of the surface. Prior studies suggest the dielectric breakdown of dielectric polymers is often initiated at the dielectric/electrode interface [42]. The injected charge from the electrodes impacts the polymers, causing the deterioration of the polymer and dielectric breakdown of the dielectrics under electrical stress. Because the surface of the P(VDF-CTFE) films is damaged by the DBD treatment, the destruction of the integrity of the surface polymer chain reduces its resistance to injected charge and some defects due to defluorination and dechlorination can initiate the material breakdown process faster. It is understandable that the breakdown field can be reduced in the treated films under the influence of charge injection. The injected charge can be electron or hole. Since the reduction in the breakdown field is observed only in the film whose treated surface is connected to the high potential end of the power supply, it implies that hole injection is a predominant process initiating the dielectric breakdown of P(VDF-CTFE) films [43]. This conclusion is consistent with those obtained in the studies of other PVDF-based polymers, such as PVDF-based terpolymers [14]. We also observe that the dielectric breakdown is reduced more significantly in the film with a rougher surface. This can be explained by the greater field concentration on a rougher surface, which causes more severe impact damage of the polymer chains by injected holes under a high applied electric field [44,45,46].

## 4. Conclusions

In summary, DBD (dielectric barrier discharge) treatment was used to modify the surface of P(VDF-CTFE) films, and the effect of the treatment on the dielectric breakdown field was studied. We found that the dielectric field of the treated film is dependent on which surface is connected to the high potential side of the high voltage power supply used for the breakdown test. A significant reduction in the breakdown field is observed when the surface subjected to DBD treatment is connected to the high potential side of the power supply for the breakdown test, and the breakdown field almost does not change if the treated surface is connected to the low potential end. These results indicate that the breakdown of the P(VDF-CTFE) film is mainly initiated by hole injection at the polymer/electrode interface. Because the DBD treatment causes damage to the polymer chains on the surface of the film, the breakdown field is reduced when the treated film is connected to the high potential side during the breakdown test. This work presents an important experimental study towards the understanding of the breakdown mechanisms in P(VDF-CTFE) polymer.

## Figures and Tables

**Table 1:** The dielectric breakdown strength of the pristine P(VDF-CTFE) film and the film treated under different conditions (Unit: MV/m).

Treatment Conditions	P(VDF-CTFE)	8 kV 60 s	10 kV 15 s	10 kV 60 s
Treated surface connected to low potential side	473.97	480.67	473.95	483.47
Treated surface connected to high potential side	473.97	495.58	319.36	442.07

**Table 2:** The element content of P(VDF-CTFE) and the films treated by DBD.

Elements	F	Cl	O	C
P(VDF-CTFE)	24.08%	1.17%	0.78%	73.97%
8 kV 60 s	22.61%	1.09%	1.55%	74.75%
10 kV 15 s	14.56%	0.72%	3.18%	81.54%
10 kV 60 s	13.47%	0.40%	4.75%	81.38%

**Table 3:** The surface roughness (Ra) of P(VDF-CTFE) and films treated by DBD (Unit: nm).

Treatment Conditions	P(VDF-CTFE)	8 kV 60 s	10 kV 15 s	10 kV 60 s
Ra	22.4	25.3	87.7	30.3

## References

[1] Prateek, Thakur V.K., Gupta R.K. (2016). Recent progress on ferroelectric polymer-based nanocomposites for high energy density capacitors: Synthesis, dielectric properties, and future aspects. Chem. Rev..

[2] Chu B.J., Zhou X., Ren K.L., Neese B., Lin M.R., Wang Q., Bauer F., Zhang Q.M. (2006). A dielectric polymer with high electric energy density and fast discharge speed. Science.

[3] Huan T.D., Boggs S., Tegssedre G., Laurent C., Cakmak M., Kumar S., Ramprasak R. (2016). Advanced polymeric dielectrics for high energy density applications. Prog. Mater. Sci..

[4] Li W.P., Jiang L., Zhang X., Shen Y., Nan C.W. (2014). High-energy-density dielectric films based on polyvinylidene fluoride and aromatic polythiourea for capacitors. J. Mater. Chem. A.

[5] Zhu L., Wang Q. (2012). Novel ferroelectric polymers for high energy density and low loss dielectrics. Macromolecules.

[6] Ranjani M., Yoo D.J., Kumar G.G. (2018). Sulfonated Fe_3_O_4_@SiO_2_ nanorods incorporated sPVdF nanocomposite membranes for DMFC applications. J. Membr. Sci..

[7] Wang Y., Zhou X., Chen Q., Chu B.J., Zhang Q.M. (2010). Recent development of high energy density polymers for dielectric capacitors. IEEE Trans. Dielectr. Electr. Insul..

[8] Leroux F., Campagne C., Perwuelz A., Gengembreb L. (2008). Polypropylene film chemical and physical modifications by dielectric barrier discharge plasma treatment at atmospheric pressure. J. Colloid Interface Sci..

[9] Chu B.J., Neese B., Lin M.R., Lu S.G., Zhang Q.M. (2008). Enhancement of dielectric energy density in the poly (vinylidene fluoride)-based terpolymer/copolymer blends. Appl. Phys. Lett..

[10] Zhou X., Chu B.J., Neese B., Lin M.R., Zhang Q.M. (2007). Electrical energy density and discharge characteristics of a poly (vinylidene fluoride-chlorotrifluoroethylene) copolymer. IEEE Trans. Dielectr. Electr. Insul..

[11] Xia W.M., Li J.J., Zhang Z.C., Xu Z. (2010). Poly (vinylidene fluoride-chlorotrifluoroethylene)/BaTiO_3_ composites with high electrical energy density. Ferroelectrics.

[12] Jow T.R., Cygan P.J. Investigation of dielectric breakdown of polyvinylidene fluoride using AC and DC methods. Proceedings of the Conference Record of the 1992 IEEE International Symposium on Electrical Insulation.

[13] O’Dwyer J.J. (1969). Theory of Dielectric Breakdown in Solids. J. Electrochem. Soc..

[14] Chen Q., Chu B.J., Zhou X., Zhang Q.M. (2007). Effect of metal-polymer interface on the breakdown electric field of poly (vinylidene fluoride-trifluoroethylene-chlorofluoroethylene) terpolymer. Appl. Phys. Lett..

[15] Han K., Li Q., Chanthad C., Gadinski M.R., Zhang G., Wang Q. (2015). A Hybrid Material Approach Toward Solution-Processable Dielectrics Exhibiting Enhanced Breakdown Strength and High Energy Density. Adv. Funct. Mater..

[16] Tang J., Duan Y.X., Zhao W. (2010). Characterization and mechanism studies of dielectric barrier discharges generated at atmospheric pressure. Appl. Phys. Lett..

[17] Xu X.J. (2001). Dielectric barrier discharge—Properties and applications. Thin Solid Film..

[18] Eliasson B., Hirth M., Kogelschatz U. (1987). Ozone synthesis from oxygen in dielectric barrier discharges. J. Phys. D.

[19] Shao T., Long K.H., Zhang C., Yan P., Zhang S.C., Pan R.Z. (2008). Experimental study on repetitive unipolar nanosecond-pulse dielectric barrier discharge in air at atmospheric pressure. J. Phys. D Appl. Phys..

[20] Liu S.H., Neiger M. (2001). Excitation of dielectric barrier discharges by unipolar submicrosecond square pulses. J. Phys. D Appl. Phys..

[21] Shao T., Wang R.X., Zhang C., Yan P. (2018). Atmospheric-pressure pulsed discharges and plasmas: Mechanism, characteristics and applications. High Volt..

[22] Shao T., Liu F., Hai B., Ma Y.F., Wang R.X., Ren C.Y. (2017). Surface modification of epoxy using an atmospheric pressure dielectric barrier discharge to accelerate surface charge dissipation. IEEE Trans. Dielectr. Electr. Insul..

[23] Liu Y., Su C., Ren X., Fan C., Zhou W., Wang F., Ding W. (2014). Experimental study on surface modification of PET films under bipolar nanosecond-pulse dielectric barrier discharge in atmospheric air. Appl. Surf. Sci..

[24] Shao T., Sun G.S., Yan P., Wang J., Yuan W.Q., Sun Y.H., Zhang S.C. (2006). An experimental investigation of repetitive nanosecond-pulse breakdown in air. J. Phys. D Appl. Phys..

[25] Jurczuk K., Galeski A., Mackey M., Hiltner A., Baer E. (2015). Orientation of PVDF α and γ crystals in nanolayered films. Colloid Polym. Sci..

[26] Han R., Jin J., Khanchaitit P., Wang J., Wang Q. (2012). Effect of crystal structure on polarization reversal and energy storage of ferroelectric poly (vinylidene fluoride-co-chlorotrifluoroethylene) thin films. Polymer.

[27] Zhou Y., Liu J., Hu X.P., Chu B.J., Chen S.T., Salem D. (2017). Flexoelectric effect in PVDF-based polymers. IEEE Trans. Dielectr. Electr. Insul..

[28] Chen Y.X., Lu H.W., Shen Z.W., Li Z.L., Shen Q.D. (2017). Cooling rate controlled microstructure evolution through flash DSC and enhanced energy density in P (VDF–CTFE) for capacitor application. J. Polym. Sci. Pol. Phys..

[29] Khanchaitit P., Han K., Gadinski M.R., Li Q., Wang Q. (2013). Ferroelectric polymer networks with high energy density and improved discharged efficiency for dielectric energy storage. Nat. Commun..

[30] Wang R., Zhang C., Liu X., Xie Q., Yan P., Shao T. (2015). Microsecond pulse driven Ar/CF_4_ plasma jet for polymethylmethacrylate surface modification at atmospheric pressure. Appl. Surf. Sci..

[31] Morent R., Geyter N.D., Leys C., Gengembre L., Payen E. (2008). Comparison between XPS- and FTIR-analysis of plasma-treated polypropylene film surfaces. Surf. Interface Anal..

[32] Bodas D.S., Mandale A.B., Gangal S.A. (2005). Deposition of PTFE thin films by RF plasma sputtering on<1 0 0> silicon substrates. Appl. Surf. Sci..

[33] Briggs D. (1998). Surface Analysis of Polymers by XPS and Static SIMS.

[34] Tong W., Lu C., Cai Y., Huang Y. (2007). Surface Modification of Fluororubber Using Atmospheric Pressure Dielectric Barrier Discharge (DBD). Plasma Sci. Technol..

[35] Károly Z., Klébert S., Al-Maliki H., Pataki T. (2016). Comparison of NPIII and DBD Plasma Treatment in Terms of Wettability of PTFE and PA6. Sci. Bull. Ser. C.

[36] Wang S.F., Li J., Suo J.P., Luo T.Z. (2010). Surface modification of porous poly (tetrafluoraethylene) film by a simple chemical oxidation treatment. Appl. Surf. Sci..

[37] Ru L., Jie-Rong C. (2006). Studies on wettability of medical poly (vinyl chloride) by remote argon plasma. Appl. Surf. Sci..

[38] Shao T., Zhang C., Long K.H., Zhang D.D., Wang J., Yan P., Zhou Y.X. (2010). Surface modification of polyimide films using unipolar nanosecond-pulse DBD in atmospheric air. Appl. Surf. Sci..

[39] Kim K.B., Tak Y.H., Han Y.S., Baik K.H., Yoon M.H., Lee M.H. (2003). Relationship between surface roughness of indium tin oxide and leakage current of organic light-emitting diode. Jpn. J. Appl. Phys..

[40] Hariprasad R., Vinothkannan M., Kim A.R., Yoo D.J. (2019). SPVdF-HFP/SGO nanohybrid proton exchange membrane for the applications of direct methanol fuel cells. J. Dispers. Sci. Technol..

[41] Vinothkannan M., Kim A.R., Nahm K.S., Yoo D.J. (2016). Ternary hybrid (SPEEK/SPVdF-HFP/GO) based membrane electrolyte for the applications of fuel cells: Profile of improved mechanical strength, thermal stability and proton conductivity. RSC Adv..

[42] Mizutani T. (1994). Space charge measurement techniques and space charge in polyethylene. IEEE Trans. Dielectr. Electr. Insul..

[43] IEDA M. (1980). Dielectric breakdown process of polymers. IEEE Trans. Dielectr. Electr. Insul..

[44] Park Y.W., Inagaki N. (2003). Surface modification of poly (vinylidene fluoride) film by remote Ar, H_2_, and O_2_ plasmas. Polymer.

[45] Du B.X., Liu H.J., Liu Y. (2007). Effects of gamma-ray irradiation on dielectric surface breakdown of polybutylene polymers. IEEE Trans. Dielectr. Electr. Insul..

[46] Ohmi T., Miyashita M., Itano M., Imaoka T., Kawanabe I. (1992). Dependence of thin-oxide films quality on surface microroughness. IEEE Trans. Electron. Devices.

